# Isomerism in double-pillared-layer coordination polymers – structures and photoreactivity

**DOI:** 10.1107/S2052252518001379

**Published:** 2018-02-14

**Authors:** In-Hyeok Park, Huiyeong Ju, Kihwan Kim, Shim Sung Lee, Jagadese J. Vittal

**Affiliations:** a Department of Chemistry, National University of Singapore, 3 Science Drive 3, 117543, Singapore; b Department of Chemistry and Research Institute of Natural Science, Gyeongsang National University, Jinju, 52828, Republic of Korea

**Keywords:** cyclo­addition reactions, supra­molecular isomerism, metal–organic frameworks, MOFs, coordination polymers, single-crystal-to-single-crystal transformations, crystal engineering, crystallization and crystal growth, solid-state transformations, structural isomerism

## Abstract

The existence of isomerism in coordination polymeric structures offers opportunities to understand structure–function relationships. Herein the serendipitous isolation is reported of two isomeric double-pillared-layer coordination polymeric structures arising from two different types of carboxyl­ate bonding of benzene-1,4-di­carboxyl­ate ligands to zinc(II), which constitutes a new type of structural isomerism. The different bonding modes not only alter the shape and size of the pores, but also the nature of interpenetration and photoreactivity. Although two *trans*,*cis*,*trans*-bpeb ligands with conjugated olefin bonds are aligned in close proximity in both of the structures, one isomer undergoes a double [2 + 2] cyclo­addition reaction and the second isomer only offers an incomplete single cyclo­addition product. This work demonstrates how small changes in the structural connectivity can have an impact on the overall structural, physical and chemical properties of such materials.

## Abstract 2

This work demonstrates how small changes in the structural connectivity, arising from two different types of carboxyl­ate bonding of benzene-1,4-di­carboxyl­ate ligands to zinc(II), have an impact on the overall structural, physical and photochemical properties in two isomeric double-pillared-layer coordination polymeric structures.

## 1. Introduction   

Recent advances in the design and construction of a wide variety of highly crystalline coordination polymers (CPs) and metal–organic frameworks (MOFs) are mainly based on the self-assembly of these compounds in a one-pot crystallization process (Steed & Atwood, 2009 ▸; Ramanan & Whittingham, 2006 ▸). Researchers seek to engineer, fine tune and control the chemical composition, dimensionality, connectivity, topology, interpenetration, pore size and shape of these solid-state materials in order to vary their physical and chemical properties for various applications (Kitagawa *et al.*, 2004 ▸; Horike *et al.*, 2009 ▸; Long & Yaghi, 2009 ▸; Zhou *et al.*, 2012 ▸; Janiak, 2003 ▸; Moulton & Zaworotko, 2001 ▸; Zaworotko, 2001 ▸; Zhang *et al.*, 2008 ▸). In other words, the solid-state properties are dictated by the molecular packing, which in turn is influenced by crystallization conditions. The crystallization conditions may yield not only different polymorphs but also isomeric products. In the multi-dimensional coordination polymeric structures, various types of structural and stereoisomerism are possible that are similar to those encountered in discrete molecules, in addition to supramolecular isomerism (Moulton & Zaworotko, 2001 ▸; Zaworotko, 2001 ▸; Zhang *et al.*, 2008 ▸; Guillerm *et al.*, 2014 ▸). These isomers provide opportunities to improve our understanding of the structure–function relationship in these polymeric materials. In this respect, new types of isomerism in CPs and MOFs have been discovered to yield interesting solid properties (Blake, 2001 ▸; Barnett *et al.*, 2012 ▸; Hu *et al.*, 2012 ▸; Panda *et al.*, 2013 ▸; Poplaukhin & Tiekink, 2010 ▸; Manna *et al.*, 2008 ▸; Karmakar *et al.*, 2017 ▸).

**Figure 1:**
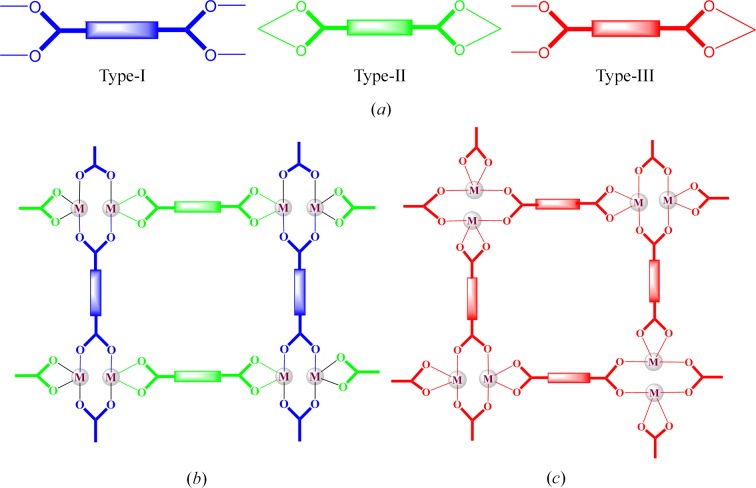
(*a*) Three types of connectivities of bdc in the chelating and bridging modes. Two structural isomers in the double-pillared-layer structure [*M*_2_(di­carboxyl­ate)_2_(pillar)_2_]. (*b*) The (4,4) ‘rectangular’ net has an idealized local *mm* symmetry with type-I and type-II linkages. (*c*) The (4,4) ‘square’ net has an idealized local fourfold rotational symmetry with type-III connectivity of di­carboxyl­ates. A rhombus-shaped net is also possible for type-III linkages. The axial positions are occupied by di­pyridyl pillar ligands, which have been omitted for clarity.

Carboxylates are known to have a variety of bonding modes to the metal centres (Guo *et al.*, 2013 ▸). Dicarboxylate spacer ligands such as the 1,4-benzenedicarboxylate anion can connect metal ions in three different ways using their chelating and bridging abilities, as shown in Fig. 1 ▸(*a*). These three types of bonding modes can generate two structural isomers while maintaining the same (4,4) connectivity as shown in Figs. 1 ▸(*b*) and 1(*c*) (Gong *et al.*, 2013 ▸; Park *et al.*, 2017 ▸). It may be noted that the isomer containing both type-I and type-II di­carboxyl­ate linkages in the [*M*_2_(di­carboxyl­ate)_2_] layer (Fig. 1 ▸ *b*) has an ideal rectangular shape with *mm* symmetry. The second isomer with type-III linkages has the shape of an ideal square with fourfold rotational symmetry in the [*M*_2_(dicarboxylate)_2_] building block, as displayed in Fig. 1 ▸(*c*).

Synthesis and isolation of these two closely related structural isomers are challenging. Furthermore, they are expected to form concomitantly in one synthesis. These two types of connectivities have been reported recently for dicarboxylates with different chemical compositions (Gong *et al.*, 2013 ▸; Park *et al.*, 2017 ▸). To the best of our knowledge, such structural isomerism has not yet been documented in metal complexes or CPs with the same chemical formula.

The influence of the single- and double-pillared-layer structures on the physical properties and chemical reactivities has been investigated (Takashima *et al.*, 2011 ▸; Mahata *et al.*, 2006 ▸; Kitaura *et al.*, 2002 ▸, 2003 ▸; Kitagawa & Matsuda, 2007 ▸; Sato *et al.*, 2010 ▸; Park, Medishetty *et al.*, 2014 ▸; Park *et al.*, 2015 ▸; Chun *et al.*, 2005 ▸; Seo *et al.*, 2009 ▸; Maji *et al.*, 2004 ▸; Henke *et al.*, 2012 ▸). Supramolecular isomers with different dimensionalities and topologies have been shown to possess different physical properties (Moulton & Zaworotko, 2001 ▸; Zaworotko, 2001 ▸). Since the two structural isomers described here have the same topology and differ only in their dicarboxylate bonding modes, it is interesting to investigate their properties. During our attempts to grow higher quality single crystals suitable for X-ray intensity data collection, we isolated a double-pillared-layer coordination polymeric structure with the molecular formula {Zn_2_(bpeb)_2_(bdc)_2_] {bdc = 1,4-benzenedicarboxylate, bpeb = 1,4-bis[2-(4-pyridyl)ethenyl]benzene}[Chem scheme1], when dimethylformamide (DMF) was used as one of the solvents. When DMF was replaced by dimethylacetamide (DMA), we were able to isolate two structural isomers formed concomitantly in a one-pot synthesis as major and minor products. Of these two isomers exhibiting twofold interpenetration, one has parallel and the other has perpendicular interpenetration with respect to the bpeb pillar ligands. As these pillar ligands have olefin bonds arranged in close proximity, it gives an opportunity to investigate the [2 + 2] cycloaddition photoreactivity in these two supramolecular isomers. Further photoluminescent properties were also recorded and discussed in this report.
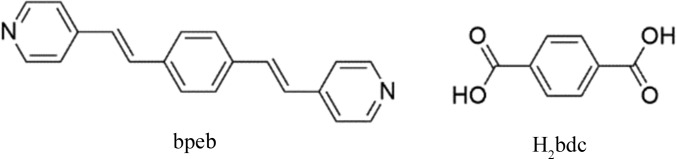


## 2. Results and discussion   

The single crystals of [Zn_2_(bpeb)_2_(bdc)_2_]·2DMF·2H_2_O·0.5DMSO (**1**) were obtained from Zn(NO_3_)_2_·6H_2_O, H_2_bdc and bpeb in a 1:1:1 molar ratio in a mixture of DMF, dimethyl sulfoxide (DMSO) and water along with a few drops of NaOH solution heated at 393 K for 48 h. When DMF was replaced by DMA, a similar solvothermal synthesis yielded the supra­molecular isomers of [Zn_2_(bpeb)_2_(bdc)_2_]·2DMA·2H_2_O (**2**) and [Zn_2_(bpeb)_2_(bdc)_2_]·DMA (**3**) concomitantly. Plate-like yellow crystals of (**2**) were the major product, whereas pale-yellow rod-shaped crystals of (**3**) were the minor product (less than 1%) in this reaction. We were unable to obtain (**3**) in larger quantities by changing the experimental conditions. The solid-state structures determined from single-crystal X-ray diffraction techniques are described below.

**Figure 2:**
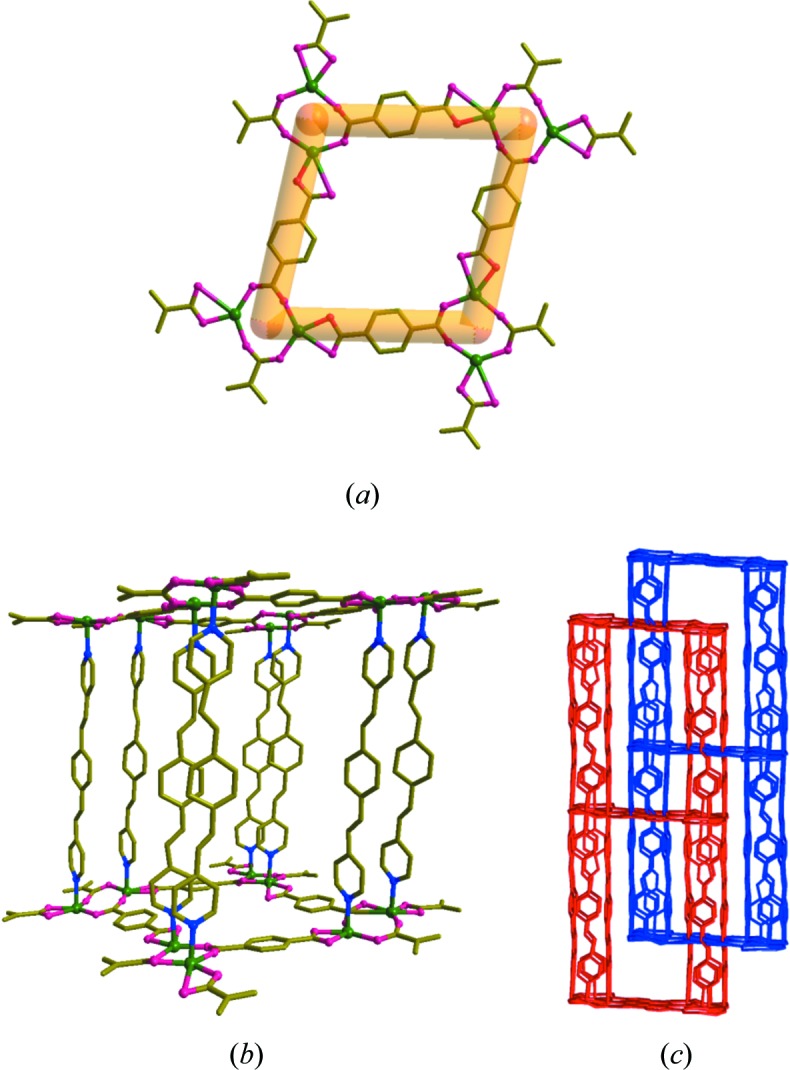
(*a*) The (4,4) net formed by Zn_2_(bdc)_2_ in (**1**). (*b*) Single **pcu** unit showing the orientations of the bpeb pillars. (*c*) Twofold parallel interpenetration of the **pcu** units. For clarity, the disorder and hydrogen atoms are not shown.

The asymmetric unit of (**1**), which crystallized in *P*2_1_/*c* with Z = 4, has a formula unit in which both bpeb ligands are disordered, see Fig. 2 ▸(*a*). Interestingly, each bpeb ligand has both *trans*,*trans*,*trans* (all-*trans*) and *trans*,*cis*,*trans* conformations in the ratio of 60:40, respectively. The dinuclear repeating unit consists of two Zn^II^ atoms bridged by two carboxyl­ate groups; each Zn^II^ is chelated by a carboxylate group (Fig. 2 ▸ *b*). The bridging carboxyl­ates have a *syn-anti*-μ_2_-η^1^:η^1^ bonding mode observed from the two sets of Zn—O—C angles [128.9 (3)° and 128.4 (3)°; 150.2 (3)° and 148.1 (3)°]. The [Zn_2_(O_2_C—C)_2_] is roughly planar. The *exo*-carboxylate groups in the *para* positions of the bdc ligands are connected to generate a (4,4) layer structure of [Zn_2_(bdc)_2_]. In fact, the Zn—bpeb—Zn distance and the diagonal distances between the centres of the Zn_2_ dimer in the Zn_2_(bdc)_2_ rhomboidal ring are the *a*, *b* and *c* unit-cell lengths. Furthermore, a closer examination reveals that each bdc has type-III carboxylate bonding. The (4,4) grid is rhombus-shaped as a result of the symmetrical bonding, with the dimensions 12.530 × 12.554 Å and an angle of 79°. The axial positions of the highly distorted octahedral Zn^II^ centres are occupied by the nitrogen atoms of the bpeb ligands. The bpeb ligands are acting like pillars, connecting the [Zn_2_(bdc)_2_] layers through pyridyl groups to produce double-pillared-layer structures with primitive cubic unit (**pcu**) topology (Fig. 2 ▸ *c*). The structure is doubly interpenetrated as a result of the large empty space. It may be noted that a pair of bpeb pillars from the neighbouring **pcu** unit penetrate each rhomboidal ring in the [Zn_2_(bdc)_2_] layer, thus forming a twofold parallel interpenetration as displayed in Fig. 2 ▸(*c*). The empty cavity generated by the bpeb ligands is filled by the double interpenetration. The total potential solvent area volume in (**1**), calculated using *PLATON* (Spek, 2009 ▸), is 1633 Å^3^, which is 26.8% of the unit-cell volume 6094.6 Å^3^.

Compound (**2**) was the major product of the crystallization using DMA in the solvothermal reaction and crystallized in the orthorhombic space group *Pcca* (No. 54) with *Z* = 4 (Fig. S2 in the supporting information). The asymmetric unit has half of the formula unit, *i.e.* [Zn(bpeb)(bdc)]. In this structure, the middle ring (C8–C15) of the bpeb is disordered and hence a mixture of all-*trans* and *trans*,*cis*,*trans* conformations are present (Fig. S2*a*). Otherwise, the structural description is very similar to that of (**1**) with type-III bonding of the bdc ligand as well as twofold parallel interpenetration (Fig. S2*d*). However, the Zn—bpeb—Zn distance and the diagonal distances between the centres of the Zn_2_ dimer in the Zn_2_(bdc)_2_ rhomboidal ring are the *b*, *a* and *c* unit-cell lengths. The total potential solvent area volume in (**2**), calculated using *PLATON* (Spek, 2009 ▸), is 1912 Å^3^, which is 30.6% of the unit-cell volume 6247.5 Å^3^.

**Figure 3:**
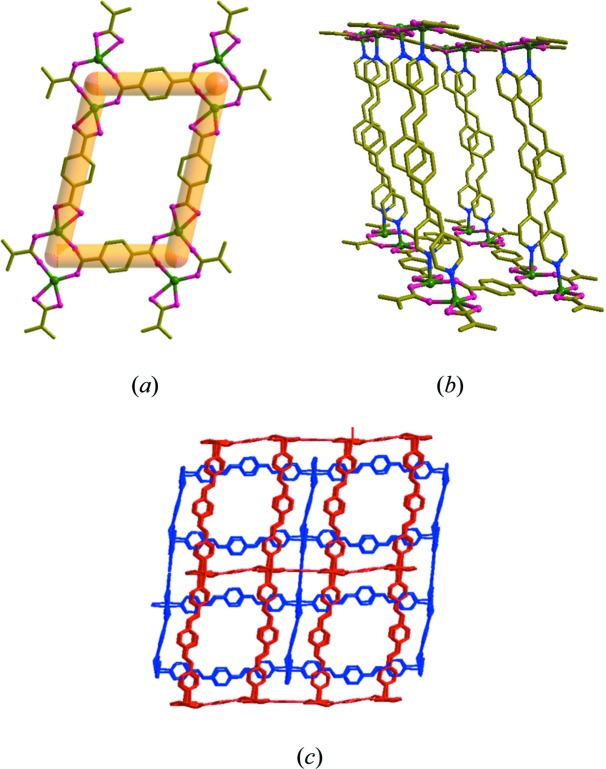
(*a*) The (4,4) net formed by Zn_2_(bdc)_2_ in (**3**). (*b*) Single **pcu** unit showing the orientations of the bpeb pillars. (*c*) Twofold perpendicular interpenetration of the **pcu** units. For clarity, the hydrogen atoms are not shown.

The minor product (**3**) crystallized in the monoclinic space group *C*2*/c* with Z = 8 (Fig. 3 ▸). The asymmetric unit contains the building unit [Zn_2_(bpeb)_2_(bdc)_2_]. In this ‘no disorder’ structure, both bpeb have *trans*,*cis*,*trans* conformations (Fig. 3 ▸ *b*). Furthermore, two types of bdc bonding, namely type-I and type-II, are present in the [Zn_2_(bdc)_2_] layer (Fig. 3 ▸ *a*), resulting in a parallelogram-shaped (4,4) grid with the dimensions 14.948  × 10.159 Å and an angle of 77.1°. The overall connectivity has **pcu** topology (Fig. 3 ▸ *c*) similar to (**1**) and (**2**). This also has twofold interpenetration, but the [Zn_2_(bpeb)_2_(bdc)_2_] units are normal with respect to each other as shown in Fig. 3 ▸(*c*). This could be attributed to insufficient space for the two bpeb pillars to interpenetrate the (4,4) net. On the other hand, the void formed by Zn—bpeb—Zn and Zn-(type-II) bdc—Zn with the dimensions 20.127 × 14.948 Å is sufficient to form a twofold perpendicular interpenetration. The total potential solvent area volume in (**3**), calculated using *PLATON* (Spek, 2009 ▸), is 2769.2 Å^3^, which is 24.3% of the unit-cell volume 11 388.7 Å^3^, indicating that this structure is more efficiently packed than (**2**).

In the double-pillared-layer structures, the two Zn^II^ centres assist in bringing the axial bpeb pillar ligands closer such that the olefin pairs satisfy Schmidt’s conditions for a [2 + 2] photo­cyclo­addition reaction (Schmidt, 1971 ▸). Interestingly, the two disordered bpeb ligands in (**1**) adopt a mixture of all-*trans* and *trans*,*cis*,*trans*-conformation (Fig. 2 ▸ *b*). Nonetheless, the six-membered rings in the two bpeb ligands are aligned in a face-to-face manner with a separation in the range 3.572–4.150 Å. The centres of the two olefin pairs are also separated by a distance in the range 3.854–4.006 Å, which is congenial for double [2 + 2] cyclo­addition reactions. However, because of the presence of mixed conformations in the disordered ligands, it was difficult to predict a priori the outcome of the photoreaction under UV light.

The ^1^H NMR spectrum of solid (**1**) was collected after irradiation under UV light for 48 h and dissolution in DMSO-*d*_6_ aided by a drop of HNO_3_; it showed the appearance of cyclo­butane peaks at 4.9–4.6 and 5.3–5.20 p.p.m. along with other peaks attributed to the aromatic protons in the region 7.7–8.9 p.p.m. (Fig. S14). Although ^1^H NMR data indicated double dimerization, the nature of the product was not clear. To investigate the solid-state structure of the photo-irradiated product of (**1**), named (**4**), we attempted to obtain single crystals of product (**4**) at the end of the cyclo­addition reaction and eventually succeeded.

**Figure 4:**
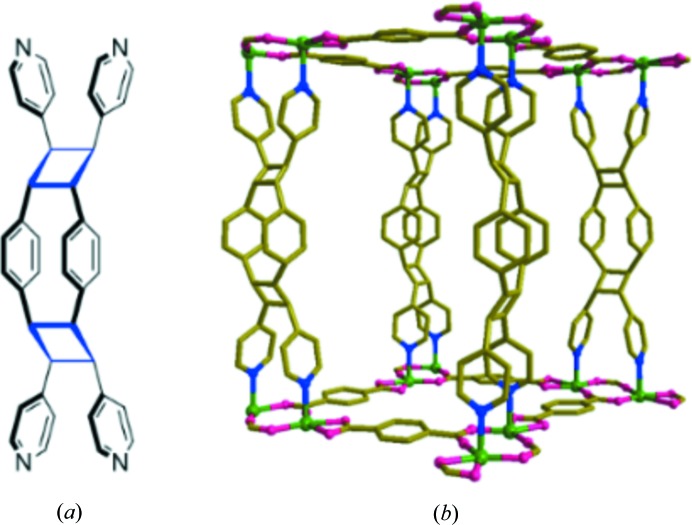
(*a*) Structure of the double-dimerized photoproduct of bpeb pairs, namely, tppcp. (*b*) A portion of the structure of (**4**) showing the double-dimerized photoproduct of *trans*,*cis*,*trans*-bpeb pairs. For clarity, the disorder and hydrogen atoms are not shown.

The single-crystal X-ray crystallographic analysis of [Zn_2_(tppcp)(bdc)_2_] (**4**) [tppcp = tetrakis­(4-pyridyl)-1,2,9,10-diethano­[2.2]*para*­*cyclo*­phane, Fig. 4 ▸ *a*)] proved the quantitative photo cyclo­addition of the *trans*,*cis*,*trans*-bpeb pairs (Fig. 4 ▸ *b*). Obviously the partially disordered all-*trans* conformation had changed to the *trans*,*cis*,*trans* conformation under UV light. Interestingly the space group changed to *Pcca* with *Z* = 4 and the asymmetric unit contained half of the unit-cell formula. The double [2 + 2] cyclo­addition product tppcp is disordered due to the presence of a centre of inversion at the centre of the ligand structure. Furthermore, the Zn—(tppcp)—Zn distance and diagonal distances between the centres of the Zn_2_ dimer in the Zn_2_(bdc)_2_ rhomboidal ring are the *b*, *a* and *c* unit-cell lengths. Therefore, the packing is very similar to that of (**2**).

In (**2**), both the bpeb pairs have a disordered mixture of all-*trans* and *trans*,*cis*,*trans* conformations similar to (**1**). The olefin bonds in these bpeb pairs were separated by 3.544 and 3.728 Å, hence (**2**) is also expected to be photoreactive. The photoreactivity of (**2**) was investigated under UV light. Solid (**2**) was irradiated under UV light for 48 h; the irradiated product (**5**) was dissolved in DMSO-*d*_6_ aided by of a drop of HNO_3_ to obtain an ^1^H NMR spectrum, which showed the appearance of cyclo­butane peaks at 4.6–4.9 p.p.m. along with other peaks attributed to aromatic protons in the region 7.7–8.9 p.p.m. (Fig. S15). In order to gain more insight into the nature of the photoproduct, we attempted a single-crystal-to-single-crystal (SCSC) reaction under UV light and succeeded.

As proven by single-crystal X-ray crystallography, the space group (*Pcca*) of the photoproduct [Zn_2_(tppcp)(bdc)_2_] (**5**) was retained from (**2**). Although the quality of the structure was poor, it was found to be isotypical to that of (**4**). The quantitative photocyclo­addition of the *trans*,*cis*,*trans*-bpeb pairs was observed. It is interesting to find that both (**1**) and (**2**) crystallized in two different space groups, yet gave isotypical photoproducts (**4**) and (**5**). It is noted that the double dimerization in bpeb has been reported in both organic compounds and MOFs, but arising only from all-*trans*-bpeb pairs (Papaefstathiou *et al.*, 2005 ▸; Friščič & MacGillivray, 2003 ▸; Liu *et al.*, 2010 ▸).

Finally, the solid-state photoreactivity of (**3**) was examined under UV light. The bpeb pairs have *trans*,*cis*,*trans* conformation, arranged in an approximately face-to-face manner with an interplanar angle of 11.4°. This was also expected to be photoreactive as the olefin bonds in these bpeb pairs were separated by 3.938 and 3.927 Å. Solid (**3**) was irradiated under UV light for 48 h and the irradiated product (**6**) was treated under similar conditons to (**1**) in order to obtain an ^1^H NMR spectrum. It showed the appearance of cyclo­butane peaks at 5.08 and 4.86 p.p.m. along with other peaks attributed to aromatic protons in the region 7.7–8.9 p.p.m. (Fig. S16). There were a number of unreacted olefin groups indicated by the presence of peaks at 7.62 and 8.00 p.p.m.. It appears that (**3**) undergoes an incomplete [2 + 2] cyclo­addition reaction.

**Figure 5:**
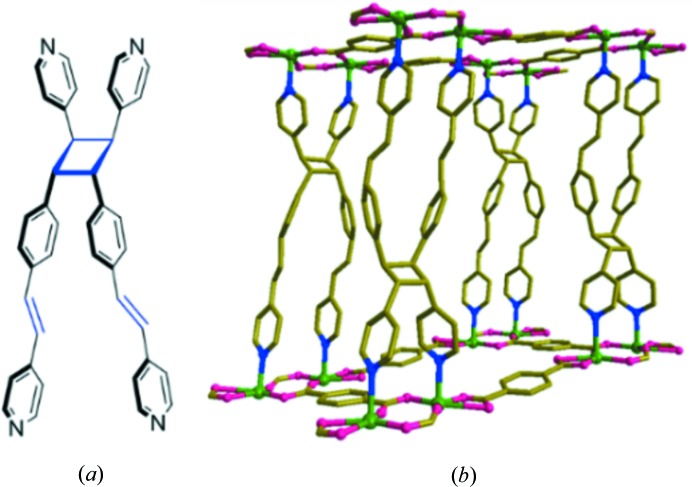
(*a*) The structural diagram of the monocyclized bpeb pairs in (**6**). (*b*) A portion of the structure of (**6**) showing the monocyclized bpeb pairs as the pillar in the photoproduct of (**3**). For clarity, the disorder and hydrogen atoms are not shown.

In order to gain more insight into the nature of the above photoproduct, we attempted an SCSC reaction under UV light. and we were able to get single crystals of the partially photodimerized product. X-ray crystallographic analysis of (**6**) shows that the cyclo­butane ring was formed between one of the two bpeb pairs, as shown in Fig. 5 ▸. Furthermore, only partial dimerization (38.9%) occurred in this crystal. Prolonged UV irradiation only destroyed the single crystals. We were unable to confirm whether complete dimerization of the single olefin pairs is possible in (**3**), resulting from a lack of suitable single crystals and the scarcity of the compound. Such photodimerization of single olefin pairs in bpeb has been reported before (Friščić & MacGillivray, 2006 ▸). It is evident that the photoreactive behaviour of (**3**) is completely different from that of (**1**) and (**2**). The packing in (**3**) is more efficient than in (**2**), as shown by the respective void volumes [30.6% in (**2**) *versus* 24.3% in (**3**). This indicates that the bpeb pairs in (**3**) do not have enough free volume to undergo double dimerization as in (**1**) or (**2**) and provides an explanation for the inability of (**3**) to remian as a single crystal after quantitative single dimerization. This is also supported by non-parallel orientations of the central phenyl­ene rings (interplanar angle, 29.9°) of the bpeb pairs in (**3**).

## 3. Conclusions   

In summary, we have serendipitously isolated two structural isomers as a result of the different bonding modes of carboxyl­ates in the bdc ligands present in the double-pillared-layer coordination polymers. We were able to synthesize one of the isomers (**1**) exclusively, but only a small quantity of the second isomer (**3**). These isomers are expected to have similar energies and are likely to be kinetic products. The MOF with diamondoid topology reported previously is also a supramolecular isomer to (**1**)-(**3**), this could be considered as the thermodynamic product (Park, Chanthapally *et al.*, 2014 ▸; Park *et al.* 2016 ▸). We have not found suitable experimental conditions to synthesize (**3**) exclusively despite many attempts. The isolation of energetically similar isomers can be compared with discovering new polymorphs of organic crystals (Haleblian & McCrone, 1969 ▸; Bernstein, 2002 ▸). These new types of structural isomers have a different packing efficiency, nature of interpenetration and photoreactivity. The two pillared *trans*,*cis*,*trans*-bpeb ligands with conjugated olefin bonds are closely aligned to each other in both structures. The isomers (**1**) and (**2**) can undergo face-to-face double [2 + 2] cyclo­addition reactions and the isomer (**3**) yields only the partial single cyclo­addition product, (**6**). These results highlight how small structural differences can influence the overall structural, physical and chemical properties. Perpendicular interpenetration observed in (**3**) seems to be more efficient for crystal packing, but this is not congenial for face-to-face double dimerization of the bpeb pairs. This work highlights the possibility of fine tuning the packing and photoreactivity of CPs and MOFs through structural isomerism.

## 4. Experimental   

### 4.1. General   

All the chemicals were reagent grade and were used without further purification. The bpeb ligand was synthesized by the reported procedure (Gutov *et al.*, 2009 ▸). Elemental analyses were carried out using a LECO CHNS-932 elemental analyser. The infrared (IR) spectra (4000–400 cm^-1^) were recorded on a Thermo Fisher Scientific Nicolet *i*S 10 F T-IR spectrometer using KBr pellets. Thermogravimetric analyses (TGA) were performed under a nitro­gen atmosphere with a heating rate of 5 K min^−1^ using a TA Instruments TGA-Q50 thermogravimetric analyser. For the TGA analysis, drying the product at 343 K for 24 h led to the loss of guest water molecules. The solid-state emission spectra were obtained from a Shimadzu RF-5301PC, using powder samples packed between glass slides in air at room temperature (296 K) using an excitation wavelength of 360 nm. Powder X-ray diffraction (PXRD) patterns were recorded on a D8 DISCOVER with GADDS (Bruker AXS) with graphite-monochromated Cu *K*α radiation (λ = 1.54056 Å) at room temperature (296 K). The UV–vis spectra were recorded on a Shimadzu UV-3600 UV-VIS-NIR spectrometer. The UV irradiation experiments were carried out on a LUZCHEM UV reactor with an 8 W dark-blue phosphor lamp (300–400 nm).

### 4.2. Preparation of [Zn_2_(bpeb)_2_(bdc)_2_]·2DMF·2H_2_O·0.5DMSO (1)   

A mixture of bpeb (20.2 mg, 0.071 mmol), H_2_bdc (12.0 mg, 0.072 mmol) and Zn(NO_3_)_2_·4H_2_O (18.6 mg, 0.071 mmol) dissolved in DMF (3 ml), H_2_O (1 ml) and DMSO (0.5 ml) were placed in a 10 ml glass tube, and then 3–4 drops of 0.1 *M* NaOH were added. The tube was sealed and kept at 393 K for 48 h, followed by cooling to room temperature (296 K) over 8 h. Pale-yellow block-shaped crystals of (**1**) suitable for X-ray analysis were obtained (yield 45%). Analysis, calculated for [C_63_H_61_N_6_O_12.5_S_0.5_Zn_2_]: C, 60.58; H, 4.92; N, 6.73; S, 1.28%; found: C, 60.45; H, 4.63; N, 6.66; S, 1.47%; IR (KBr pellet) 3447, 3051, 2926, 2838, 1676, 1605, 1501, 1429, 1385, 1257, 1220, 1132, 1092, 1067, 1017, 970, 875, 835, 750 and 659 cm^−1^.

### 4.3. Preparation of a mixture of [Zn_2_(bpeb)_2_(bdc)_2_]·2DMA ·2H_2_O (2) and [Zn_2_(bpeb)_2_(bdc)_2_]·DMA (3)   

A mixture of bpeb (19.9 mg, 0.070 mmol), H_2_bdc (11.8 mg, 0.071 mmol) and Zn(NO_3_)_2_·4H_2_O (18.8 mg, 0.072 mmol) dissolved in DMA (3 ml), H_2_O (1 ml) and DMSO (0.5 ml) were placed in a 10 ml glass tube, and then 3–4 drops of 0.1 *M* NaOH were added. The tube was sealed and kept at 393 K for 48 h, followed by cooling to room temperature (296 K) over 8 h. Pale-yellow plate-shaped crystals (**2**) (major) and pale-yellow rod-shaped crystals (**3**) (as a minor product, less than ~1% yield) suitable for X-ray analysis were obtained. For (**2**): analysis, calculated for [C_64_H_62_N_6_O_12_Zn_2_]: C, 62.09; H, 5.05; N, 6.79%; found: C, 62.15; H, 5.02; N, 7.21%; IR (KBr pellet) 3026, 2884, 2821, 1637, 1609, 1508, 1388, 1224, 1036, 954, 869, 840, 752 and 664 cm^−1^. For (**3**): IR (KBr pellet) 3033, 2895, 1631, 1543, 1388, 1237, 1073, 949, 830, 751, 715 and 668 cm^−1^. (**3**) was produced only in very low yield, hence no analytical or IR data could be obtained for this compound.

### 4.4. Preparation of [Zn_2_(tppcp)(bdc)_2_]·1.6DMF·2.8H_2_O·0.2DMSO (4)   

(**4**) was obtained by UV irradiation of the single crystals of (**1**) for 48 h. Analysis, calculated for [C_61.2_H_58_N_5.6_O_12.6_S_0.2_Zn_2_]: C, 60.71; H, 4.83; N, 6.48; S, 0.53%; found: C, 60.95; H, 4.72; N, 6.19; S, 0.49%; IR (KBr pellet) 3447, 3045, 2946, 2883, 1674, 1616, 1542, 1507, 1387, 1224, 1093, 1071, 830, 751 and 669 cm^−1^.

### 4.5. Preparation of [Zn_2_(tppcp)(bdc)_2_]·2DMA·2H_2_O (5) and [Zn_2_(bpeb)_0.6_(bpbpvpcb)_0.4_(bdc)_2_]·DMA (6)   

(**5**) and (**6**) were obtained by UV irradiation of the single crystals of (**2**) and (**3**) for 48 h, respectively. For (**5**): analysis, calculated for [C_62.8_H_61.5_N_5.7_O_12.8_Zn_2_]: C, 61.24; H, 5.03; N, 6.48; found: C, 61.11; H, 4.79; N, 6.17%; IR (KBr pellet) 3447, 3044, 2943, 1618, 1501, 1388, 1224, 1071, 1016, 928, 829, 751, 706 and 669 cm^−1^. For (**6**): IR (KBr pellet) 3051, 2932, 1605, 1524, 1375, 1237, 1073, 949, 830, 751, 715 and 668 cm^−1^. (**6**) was produced only in very low yield, hence no analytical or IR data could be obtained for this compound.

### 4.6. X-ray crystallographic analysis   

Crystal data for (**1**) were collected at 100 K and (**2**)-(**6**) were collected at 173 K on a Bruker SMART APEX II ULTRA diffractometer equipped with graphite-monochromated Mo *K*α radiation (λ = 0.71073 Å) generated by a rotating anode (Table 1 ▸). The preliminary cell parameters for the compounds were obtained from a least-squares refinement (from 36 collected frames). Data collection, data reduction and absorption correction were carried out using the software package of *APEX2* (Bruker, 2008 ▸). All of the calculations for the structure determination were carried out using the *SHELXTL* package (Bruker, 2001 ▸). Relevant crystal data collection and refinement data for the crystal structures of **(1)**–**(6)** are summarized in Table S1.

## 5. Related literature   

The following references are cited in the supporting information: Balamurugan *et al.* (2012 ▸); Elacqua *et al.* (2009 ▸); Spek (2015 ▸); Horner & Hünig (1982 ▸); Peedikakkal & Vittal (2008 ▸); Peedikakkal *et al.* (2010 ▸).

## Supplementary Material

Crystal structure: contains datablock(s) 1. DOI: 10.1107/S2052252518001379/lc5095sup1.cif

Crystal structure: contains datablock(s) 2. DOI: 10.1107/S2052252518001379/lc5095sup2.cif

Crystal structure: contains datablock(s) 3. DOI: 10.1107/S2052252518001379/lc5095sup3.cif

Crystal structure: contains datablock(s) 4. DOI: 10.1107/S2052252518001379/lc5095sup4.cif

Crystal structure: contains datablock(s) 5. DOI: 10.1107/S2052252518001379/lc5095sup5.cif

Crystal structure: contains datablock(s) 6. DOI: 10.1107/S2052252518001379/lc5095sup6.cif

Structure factors: contains datablock(s) 3. DOI: 10.1107/S2052252518001379/lc50953sup7.hkl

Structure refinements, tables, spectra and figures. DOI: 10.1107/S2052252518001379/lc5095sup8.pdf

CCDC references: 1558995, 1822407, 1558997, 1558998, 1559000, 1558999

## Figures and Tables

**Table:** 

	(**1**) CCDC 1558995	(**2**) CCDC 1558996	(**3**) CCDC 1558997
Formula	C_56_H_40_N_4_O_8_Zn_2_	C_56_H_40_N_4_O_8_Zn_2_	C_56_H_40_N_4_O_8_Zn_2_
Formula weight	1027.66	1027.66	1027.66
Temperature (K)	100	173	173
Crystal system	Monoclinic	Orthorhombic	Monoclinic
Space group	*P*2_1_/*c*	*Pcca*	*C*2/*c*
*a* (Å)	20.3028 (9)	19.490 (3)	27.5072 (12)
*b* (Å)	19.3686 (9)	20.220 (3)	29.3890 (14)
*c* (Å)	15.9389 (7)	15.854 (2)	14.9485 (7)
*β* (°)	103.500 (2)	90	109.537 (2)
*V* (Å^3^)	6094.6 (5)	6247.8 (15)	11388.7 (9)
*Z*	4	4	8
*D*_calc_(g cm^−3^)	1.120	1.093	1.199
*μ* (mm^−1^)	0.835	0.815	0.894
2*θ*_max_ (°)	52	52	52
Reflections collected	128744	49542	55236
Independent reflections	11944 (*R*_int_ = 0.0378)	5982 (*R*_int_ = 0.1052)	14223 (*R*_int_ = 0.0878)
Goodness-of-fit on *F* ^2^	1.176	1.083	1.094
*R*_1_, *wR*_2_ [*I* > 2*σ*(*I*)]	0.0719, 0.1571	0.1233, 0.3828	0.0434, 0.1265
*R*_1_, *wR*_2_ (all data)	0.0804, 0.1606	0.1673, 0.3555	0.0593, 0.1325

**Table:** 

	(**4**) CCDC 1558998	(**5**) CCDC 1558999	(**6**) CCDC 1559000
Formula	C_56_H_40_N_4_O_8_Zn_2_	C_56_H_40_N_4_O_8_Zn_2_	C_56_H_40_N_4_O_8_Zn_2_
Formula weight	1027.66	1027.66	1027.66
Temperature (K)	173	173	173
Crystal system	Orthorhombic	Orthorhombic	Monoclinic
Space group	*Pcca*	*Pcca*	*C*2/*c*
*a* (Å)	20.030 (3)	20.0663 (6)	27.430 (2)
*b* (Å)	20.005 (3)	19.9342 (7)	29.305 (2)
*c* (Å)	15.145 (2)	14.8166 (5)	14.9087 (11)
*β* (°)	90	90	107.070 (5)
*V* (Å^3^)	6068.8 (14)	5926.7 (3)	11456.2 (16)
*Z*	4	4	8
*D*_calc_(g (cm^−3^)	1.125	1.152	1.192
*μ* (mm^−1^-1^^)	0.839	0.859	0.889
2*θ*_max_ (°)	52.	52.	53.
Reflections collected	46693	64566	50356
Independent reflections	5979 (*R*_int_ = 0.0739)	5651 (*R*_int_ = 0.1189)	11878 (*R*_int_ = 0.0918)
Goodness-of-fit on *F* ^2^	1.115	1.027	1.035
*R*_1_, *wR*_2_ [*I* > 2*σ*(*I*)]	0.0731, 0.2166	0.1094, 0.3151	0.0862, 0.2151
*R*_1_, *wR*_2_ (all data)	0.0.0993, 0.2313	0.1736, 0.3466	0.1439, 0.2377
