# Supplementary material for: Isomerism in double-pillared-layer coordination polymers – structures and photoreactivity
Source: IUCrJ. 2018 Feb 14;5(Pt 2):182–9. doi: 10.1107/S2052252518001379 (PMC5947723; doi:10.1107/S2052252518001379)
Supplement: Supplementary file 8 [file m-05-00182-sup8.pdf]

IUCrJ

Volume 5 (2018)

Supporting information for article:

Isomerism in double-pillared-layer coordination polymers – structures and photoreactivity

In-Hyeok Park, Huiyeong Ju, Kihwan Kim, Shim Sung Lee and Jagadese J. Vittal

Structure Refinement Details

[$Zn_2(bdc)_2(bpeb)_2$] (1). Crystallized in $P2_1/c$ with Z = 4. The asymmetric unit contains the repeating unit of the double pillared-layer structure. The major disorders and their treatments are described here. Our initial attempt to refine the structure using the normal data was not satisfactory and hence the data were SQUEEZED. O4 was disordered with occupancies fixed at 0.6 and 0.4. Anisotropic thermal parameters were refined for these two O atoms without any restraints/constraints. The two bpeb ligands were disordered. Two disorder components were included and a common occupancy was refined to 0.605(6). Anisotropic thermal parameters were refined for all the non-hydrogen atoms in the major component. Soft constraints like FLAT SAME were used for some of the phenyl and pyridyl rings. For the non-hydrogen atoms in the minor disorder, only isotropic thermal parameter could be refined after all the options tried. Again, FLAT and SAME were used to retain a reasonably ideal geometry of few phenyl and pyridyl rings. The agreement factors are much better for this data as compared to the normal data. Unsqueezed refinements provided 1.5DMF and 3.5H_2O lattice solvents, which could be different from the analytical data.

[$Zn_2(bdc)_2(bpeb)_2$] (2). This is crystallized in the orthorhombic space group *Pcca* (No. 54) with Z = 4. The asymmetric unit contains half of the repeating unit. It was not possible to locate any of the solvents in the lattice and the agreement factors were still unreasonably high (R_1 = 0.2065) and hence we have Squeezed the data. The bpeb ligand was disordered. Only the disorder component in the middle of the ligand (C8 to C15) was resolved. Two models were included and refined. Hard constraints were used to hold the geometry close to ideal. Only common isotropic thermal parameters were refined for each group of disorder. A common occupancy factor of the two disorder components was refined to 0.611(17). Interesting to note that the major component has *trans,trans,trans* conformation and the minor disorder component has *trans,cis,trans* conformation. From these data only the connectivity could be confirmed beyond any doubt and the bond distances and angles should be treated with caution.

[$Zn_2(bdc)_2(bpeb)_2$] (3). After several attempts, an excellent crystallographic datan were collected for this compound. Initially normal data were used to refine the model. Surprisingly no disorders were present in in the two bpeb ligand. 1.5 DMA molecules were located in two different regions and refined in the model. Scattered electron densities were assigned to O atoms of the water molecules (1 × 0.5 and 4 × 0.25). For uniformity with other crystal data, we used squeezed out the guest solvents in the lattice and kept the final model with squeezed data (Spek, 2015). The final model was refined to R_1 = 0.0434 and wR_2 = 0.1265 for 10607 data with I > 2σ(I) and 631 parameters and R_1 = 0.0593 and wR_2 = 0.1325

for all 14223 data. Anisotropic thermal paramters were refined for all the non-hydrogen atoms and restraints or constraints were imposed in no disordered structure.

[$Zn_2(bdc)_2$(bpeb-dimer)] (4). Crystallized in the orthorhombic space group *Pcca* with $Z = 4$. The asymmetric unit contains half of the formula unit. The dimer formed by dimerization of *trans,cis,trans*-bpeb disordered due to crystallographic centre of inversion. Due to this disorder soft constraints (FLAT, SAME, SADI) were used to retain the geometry of molecule. Only isotropic thermal parameter could be refined for these bpeb-dimer fragments. Again one DMF was disordered by crystallographic inversion centre. The rest of the electron densities were too complicated to resolve. Hence they were assigned as O atoms of water molecules. They could as well be highly disordered DMF. The formula was fixed at [$Zn_2(bdc)_2$(bpeb-dimer)]·DMF·6.5H_2O. The final agreement factors are $R_1 = 0.0839$, wR_2 = 0.2392 & GoF = 1.049 for 5979 data and 371 parameters. For uniformity, the data were squeezed (Spek, 2015). The final agreement factors for squeezed data are $R_1 = 0.0731$, wR_2 = 0.2166 & GoF = 1.115 for 5979 data and 294 parameters.

[$Zn_2(bdc)_2$(bpeb-dimer)] (5). Crystallized in the orthorhombic space group *Pcca* with $Z = 4$. The The asymmetric unit contains half of the formula unit. The dimer formed by dimerization of *trans,cis,trans*-bpeb disordered due to crystallographic centre of inversion. This is isotypical to **4**. For the normal data with $R_{int} = 0.1189$, no solvent molecules could be identified and hence it was squeezed. Otherwise, the refinements were also similar to **4** but the final agreement factors, thermal parameters were not very good. Nevertheless, the connectivity is proved beyond any doubt to show that this is completely dimerized product of *trans,cis,trans*-bpeb pairs.

[$Zn_2(bdc)_2$(bpeb-dimer)] (6). The asymmetric unit contains the whole repeating unit in C_2/c with Z = 8. Initially refinement was attempted with normal data with $R_{int} = 0.1078$. Due to high agreement factors and problem in completing satisfactory refinements of the structural model, SQUEEZEd data was used. The refinement is marred by the disorder created by the partial dimerization. Only one of the two olefin bonds was found to form cyclobutane ring. The dimerized fragment (C8a - C40a) was included in the refinement. A common occupancy factor was refined to 0.610(6). Only isotropic thermal parameters were refined for these "disordered" fragments, as anisotropic refinement failed. Even though the geometry of the rings were confined to hexagon using FLAT, SADI and SAME options they were not strictly maintained in some cases. However, the agreement factors are better than the unsqueezed data (shown below) and hence reported here. Although the bond distances and angles are not very accurate for a detailed comparison, partial single dimerization photoreaction is proved beyond any doubt. Solvent accessible void from PLATON program is 2533 Å^3 and 22.1%.

The unsqueezed data gave half DMF was found and refined in the structure. The model with normal data was refined to $R_1 = 0.1028$ and wR_2 = 0.3049, GooF = 1.087 for 6671 > 4sig (Fo) and 629 parameters and 176 staints. For all 11878 data $R_1 = 0.1751$.

Table S1. Voids volume and percentage of voids volume in **1-6**.

**Xtl**	**Void Vol/Total Vol, Å^3**	**%**	**Xtl**	**Void Vol/Total Vol, Å^3**	**%**
**1**	1633/6094.6	26.8	**4**	1748.3/6068.8	28.8
**2**	1912/6247.5	30.6	**5**	1690/5926.7	28.5
**3**	2769.2/11388.7	24.3	**6**	2533/11456.2	22.1

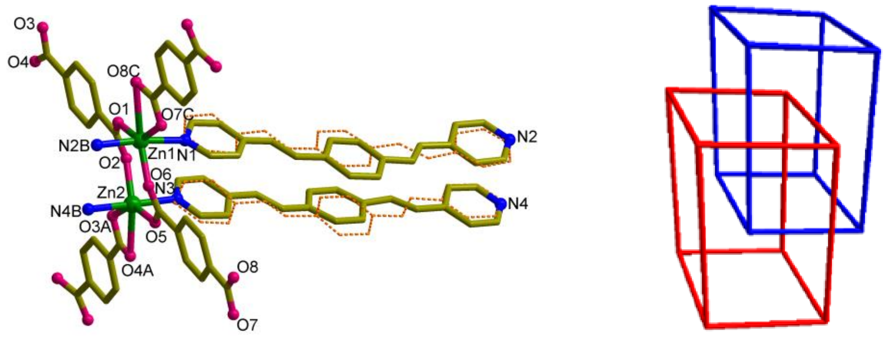

(a) (b)

Figure S1 (a) Building block in **1** showing the disordered bpeb ligands. Symmetry codes: (A) x, 1.5-y, 0.5+z; (B) 1+x, y, z; (C) x, 0.5-y, -0.5+z. (**b**) **pcu** topology with interpenetration in **1**.

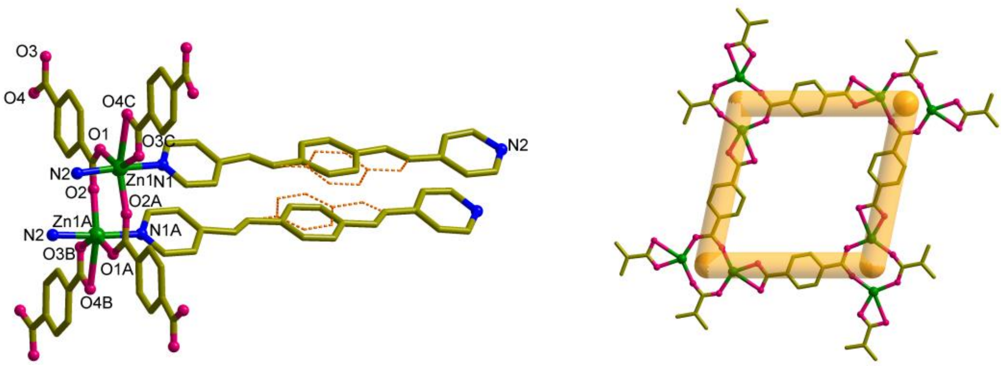

(a) (b)

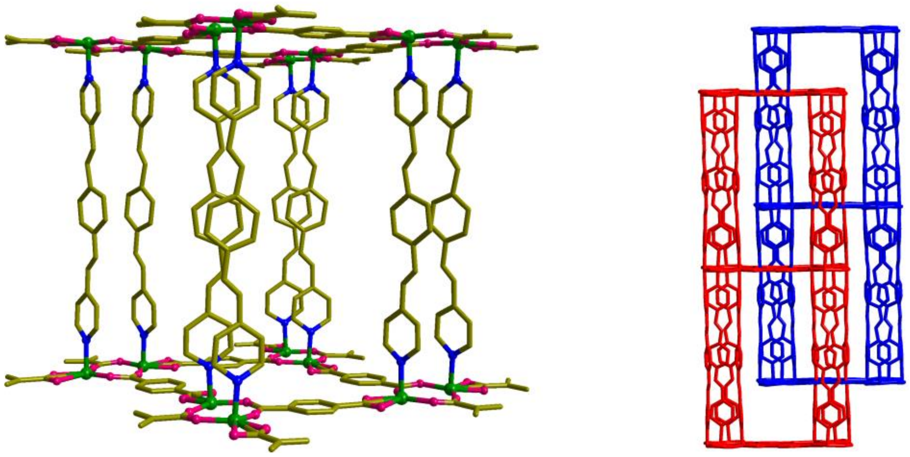

(c) (d)

Figure S2 (a) Building block in **2** showing the disordered bpeb ligands. Symmetry codes: (A) 1-x, y, 0.5-z; (B) 1.5-x, y, -0.5+z; (C) -0.5+x, y, 1-z. (b) The (4,4) net formed by $Zn_2(bdc)_2$. (c) Single **pcu** unit showing the orientations of the bpeb pillars in **2**. (d) Two-fold *parallel* interpenetration of the **pcu** units.

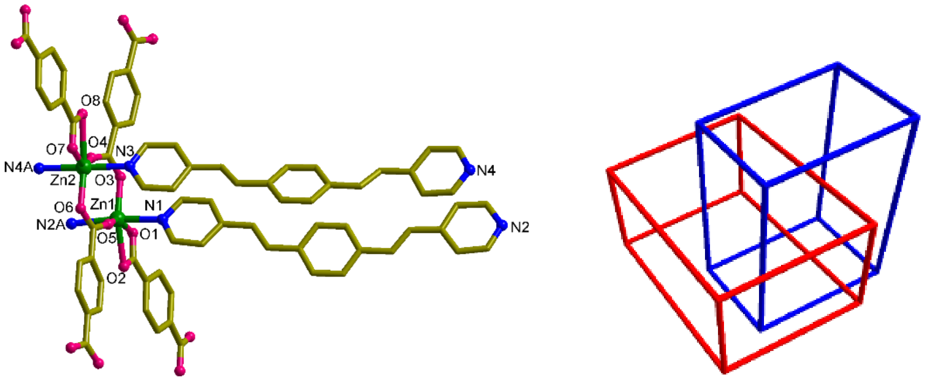

(a) (b)

Figure S3 (a) Building block in **3**. Symmetry code: -0.5+x, -0.5+y, z. There is no disorder observed in this structure. (b) Two-fold *perpendicular* interpenetration of the **pcu** units.

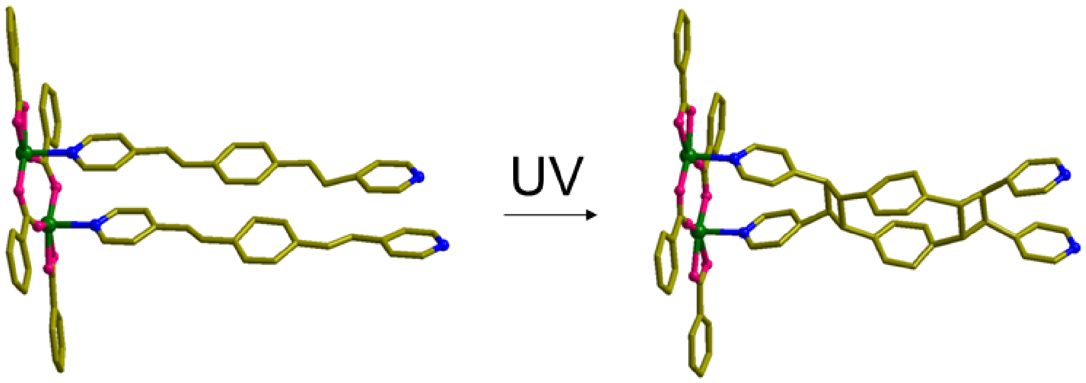

(a)

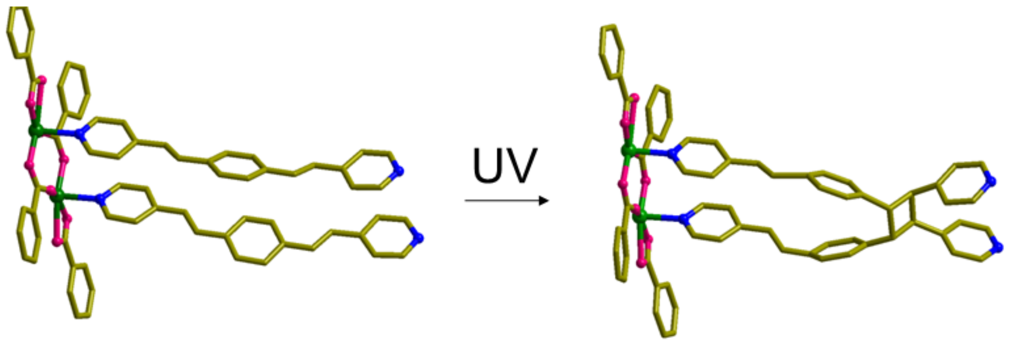

(b)

Figure S4 Schematic diagrams of the photoreactive behaviour of the two structural isomers. (a) Face-to-face double dimerization of **1** and **2**. (b) Partial single cyclization of olefin pairs in **3** under UV radiation.

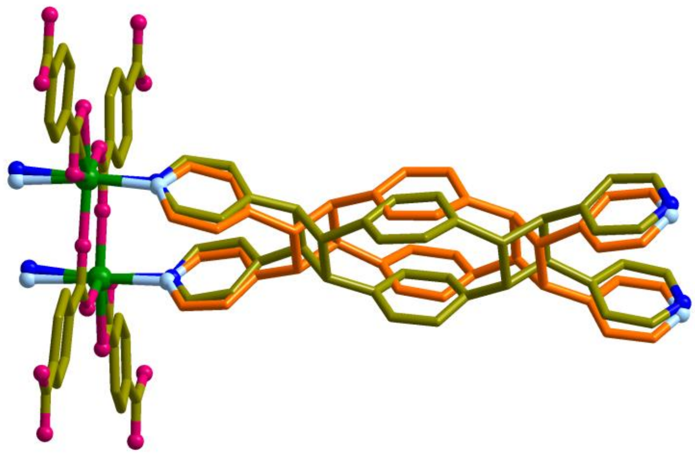

Figure S5 Building block in **4** showing the disordered bpeb-dimer ligands.

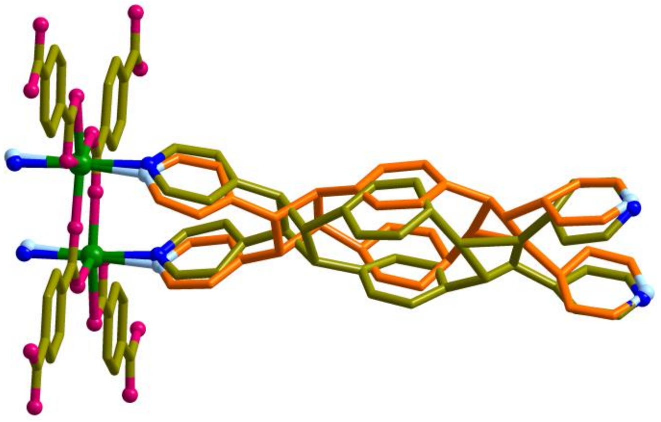

Figure S6 Building block in **5** showing the disordered bpeb-dimer ligands.

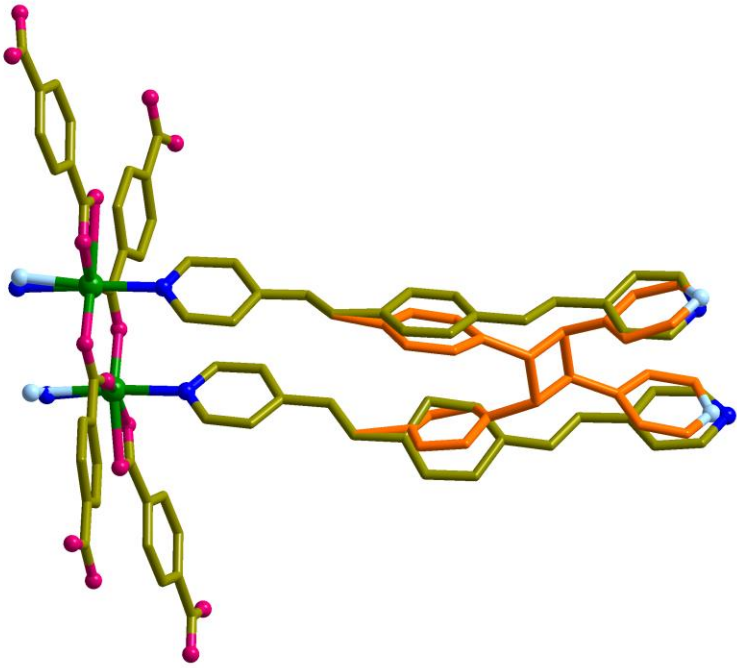

Figure S7 Building block in **6** showing the disordered bpeb and bpeb-dimer ligands.

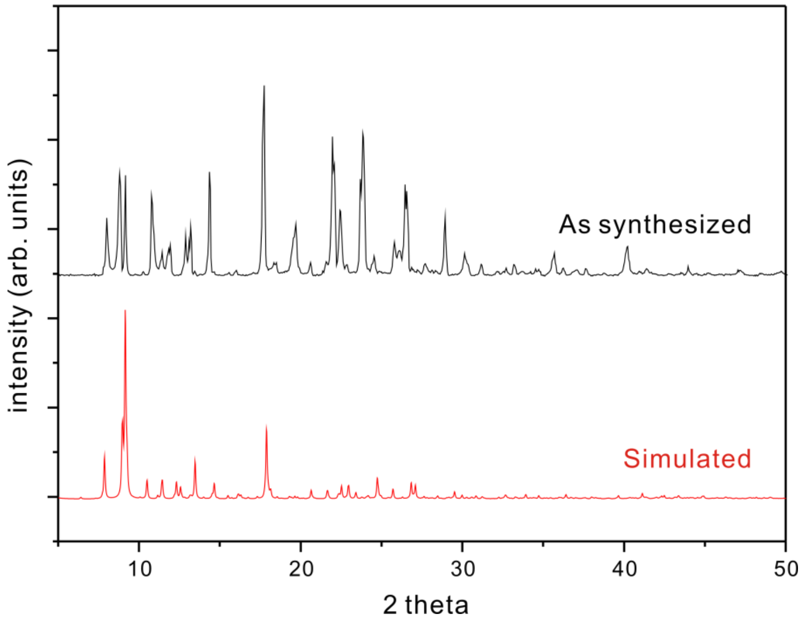

Figure S8 PXRD patterns for **1**: (top) as synthesized and (bottom) simulated from the single crystal X-ray data. The discrepancies in the intensities may be due to preferred orientations of the powder or due to partial removal of solvents during grinding.

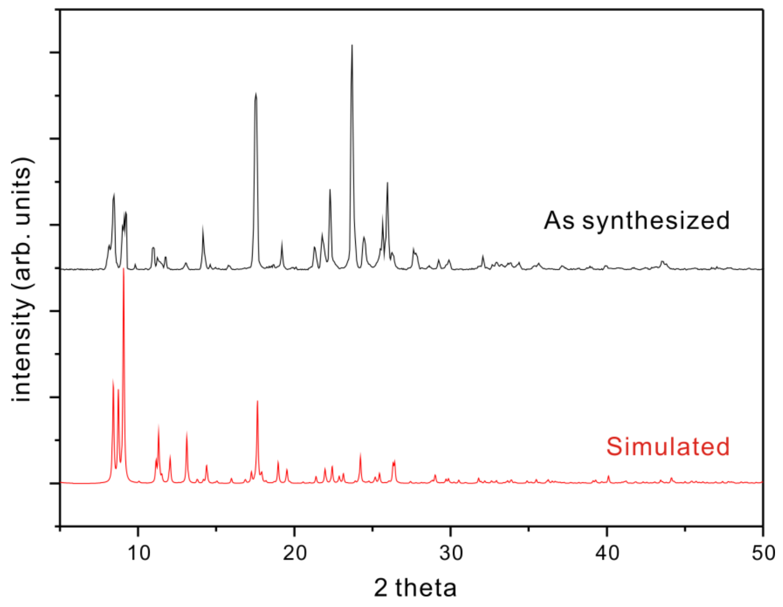

Figure S9 PXRD patterns of **2**: (top) as synthesized and (bottom) simulated from the single crystal X-ray data. The discrepancies in the intensities may be due to preferred orientations of the powder or due to partial removal of solvents during grinding.

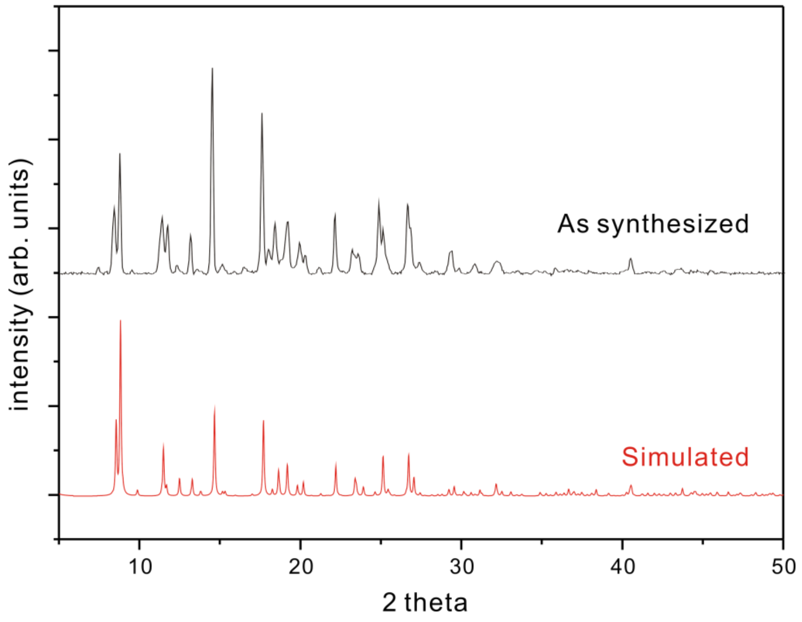

Figure S10PXRD patterns of **4**: (top) as synthesized and (bottom) simulated from the single crystal X-ray data. The discrepancies in the intensities may be due to preferred orientations of the powder or due to partial removal of solvents during grinding.

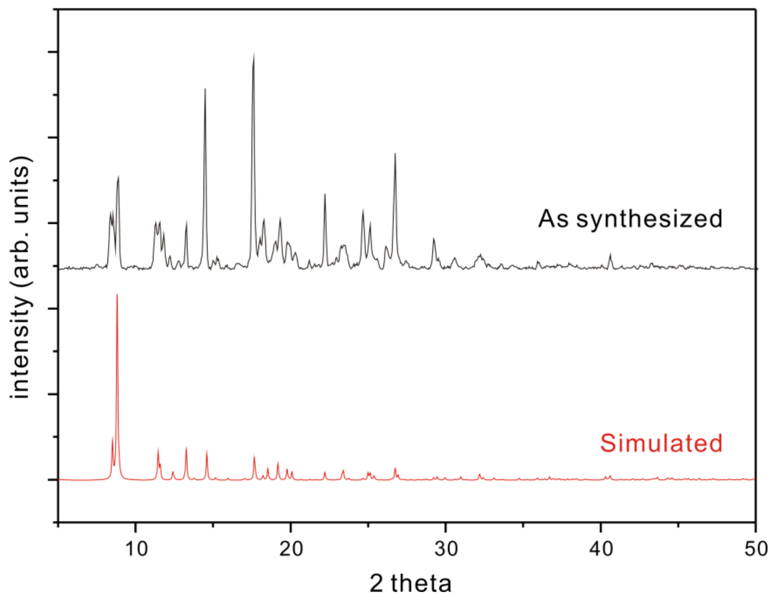

Figure S11PXRD patterns of **5**: (top) as synthesized and (bottom) simulated from the single crystal X-ray data. The discrepancies in the intensities may be due to preferred orientations of the powder or due to partial removal of solvents during grinding.

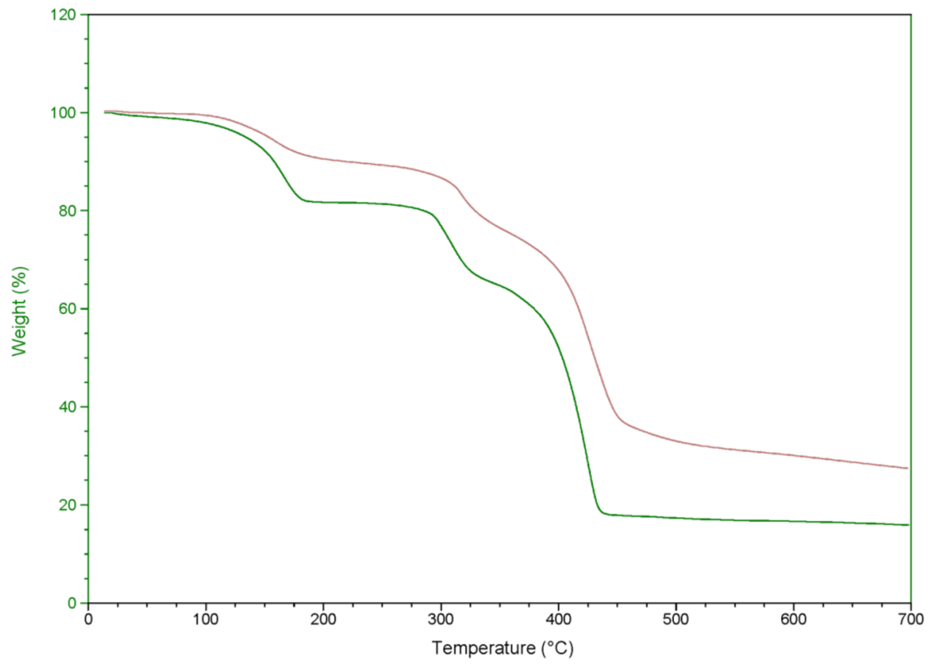

Figure S12TGA curve of **1** (green line) and **4** (red line) with heating rate of 5°C·min^{-1} under N_2 flow. The desolvated **1** and **4** are stable up to 280 and 300°C, respectively.

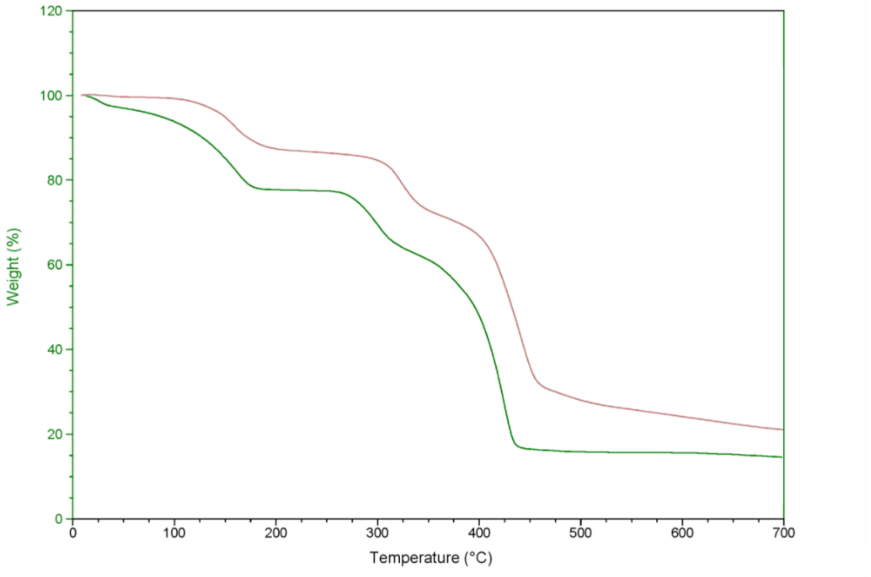

Figure S13 TGA curve of **2** (green line) and **5** (red line) with heating rate of 5°C·min^{-1} under N_2 flow. The desolvated **2** and **4** are stable up to 280 and 300°C, respectively.

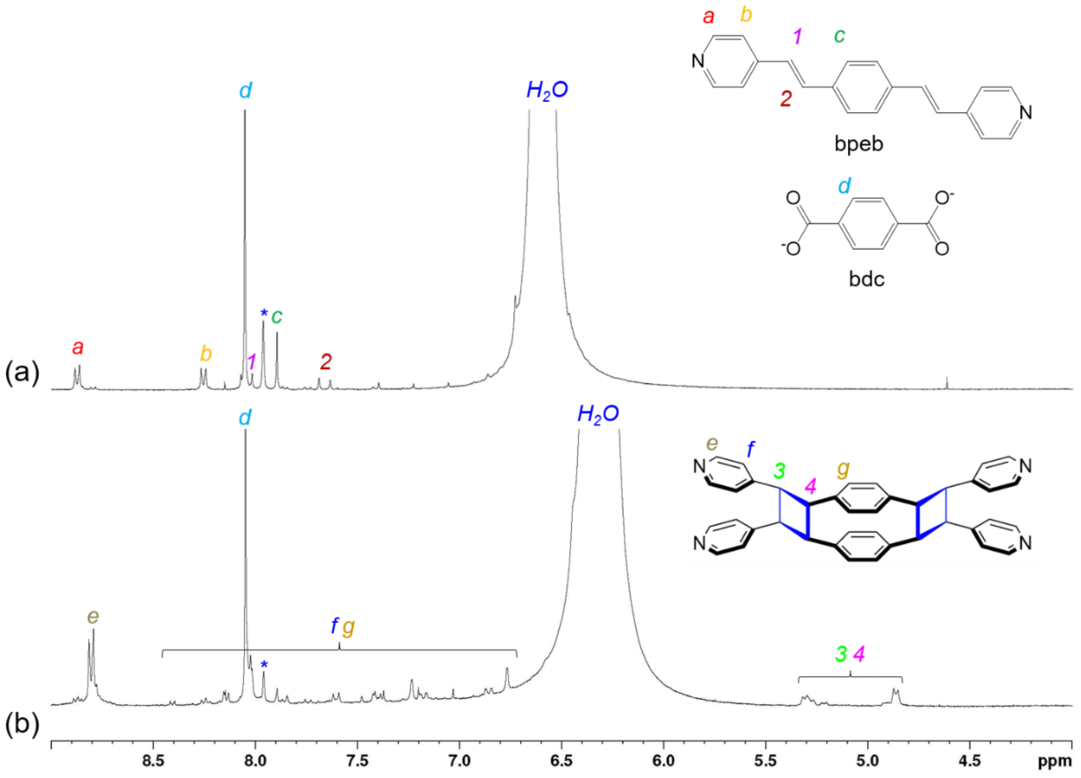

Figure S14 The ^{1}H NMR spectra of (a) **1** and (b) **4** in DMSO-d_6 with a small drop of HNO_3 to dissolve the crystals. The humps around 6.6 ppm and 6.4 ppm are due to the protonated water in **1** and **4**, respectively. During the dissolution of the highly insoluble MOFs with HNO_3, we suspect the highly strained cyclobutanes undergo isomerization process. Such isomerization of cyclobutanes are well-known in the literature (Horner & Hünig (1982), Peedikakkal & Vittal (2008) and Peedikakkal et al (2010))

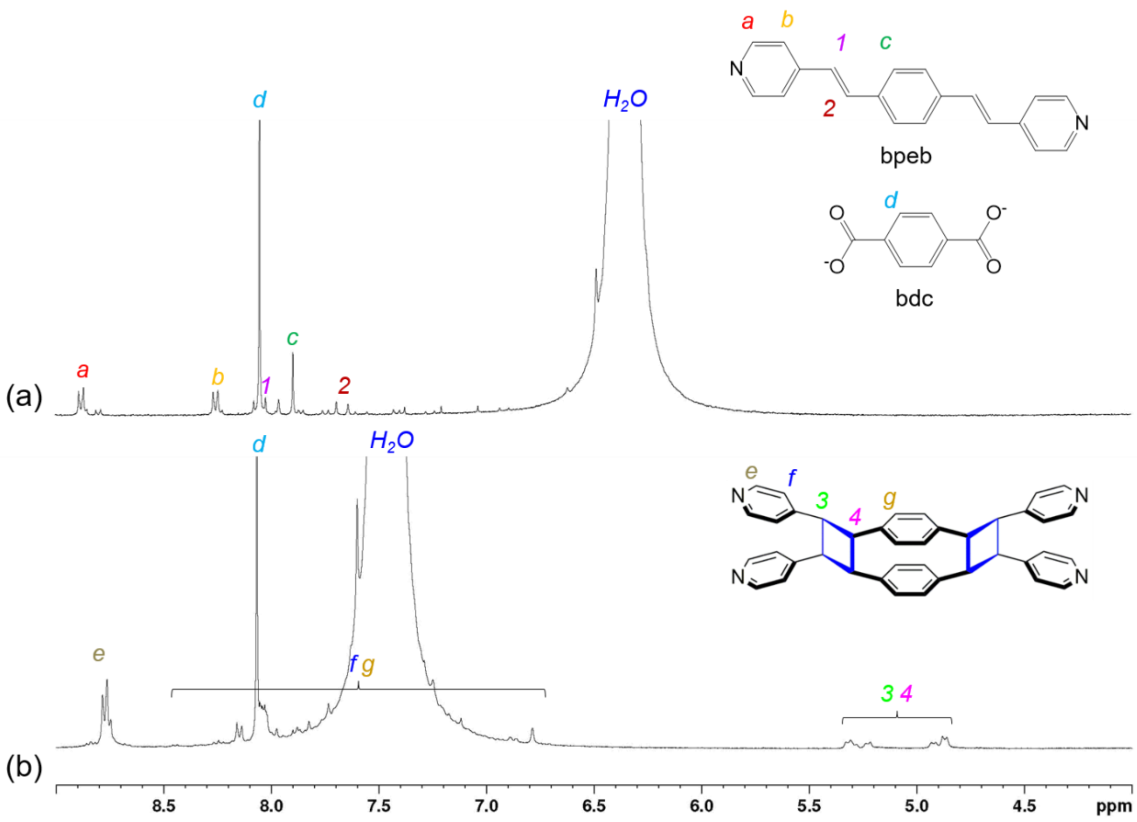

Figure S15 The ^{1}H NMR spectra of (a) **2** and (b) **5** in DMSO-d_6 with a small drop of HNO_3 to dissolve the crystals. The humps around 6.4 ppm and 7.4 ppm are due to the protonated water in **2** and **5**, respectively. During the dissolution of the highly insoluble MOFs with HNO_3, we suspect the highly strained cyclobutanes undergo isomerization process. Hence more species are formed than expected unlike *trans*,*trans*,*trans*-bpeb pairs. Such isomerization of cyclobutanes are well-known in the literature (Horner & Hünig (1982), Peedikakkal & Vittal (2008) and Peedikakkal et al (2010))

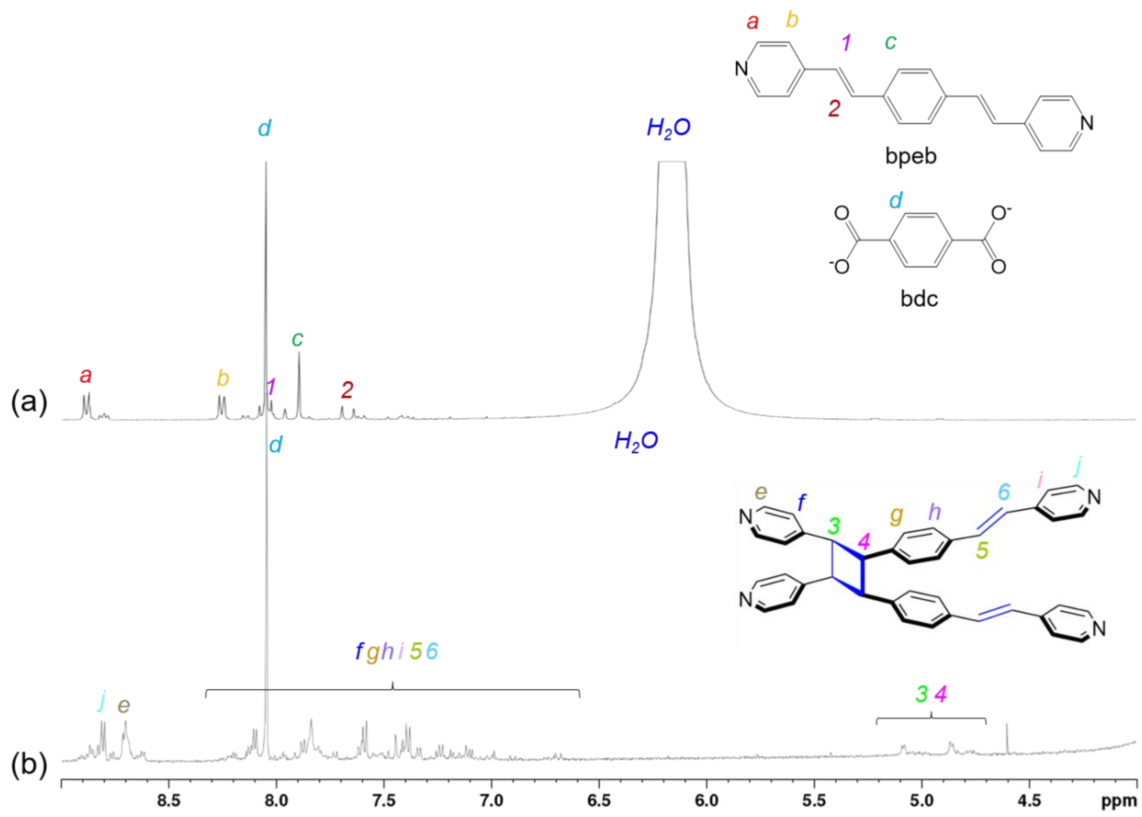

Figure S16 The ^{1}H NMR spectra of (a) **3** and (b) **6** in DMSO-d_6 with a small drop of HNO_3 to dissolve the crystals. The humps around 6.4 ppm and 7.4 ppm are due to the protonated water in **3** and **6**, respectively. During the dissolution of the highly insoluble MOFs with HNO_3, we suspect the highly strained cyclobutanes undergo isomerization process. Hence more species are formed than expected unlike *trans*,*trans*,*trans*-bpeb pairs. Such isomerization of cyclobutanes are well-known in the literature (Horner & Hünig (1982), Peedikakkal & Vittal (2008) and Peedikakkal et al (2010))

The solid-state photoluminescence (PL) spectra of **1, 2, 4** and **5** were recorded upon excitation at 365 nm. Both **1** and **2** exhibit green emission centred at λ_{max} at 515 and 507 nm (Fig. S17). Whereas the PL emission show hypsochromic shift for **4** and **5**. This is attributed to the disruption of the delocalization of electrons in bpeb ligands due to the dimerization of the olefin pairs (Balamurugan, 2012). However, red shift of the PL in the double dimerized photoproduct of bpeb was reported by MacGillivray (Elacqua, 2009).

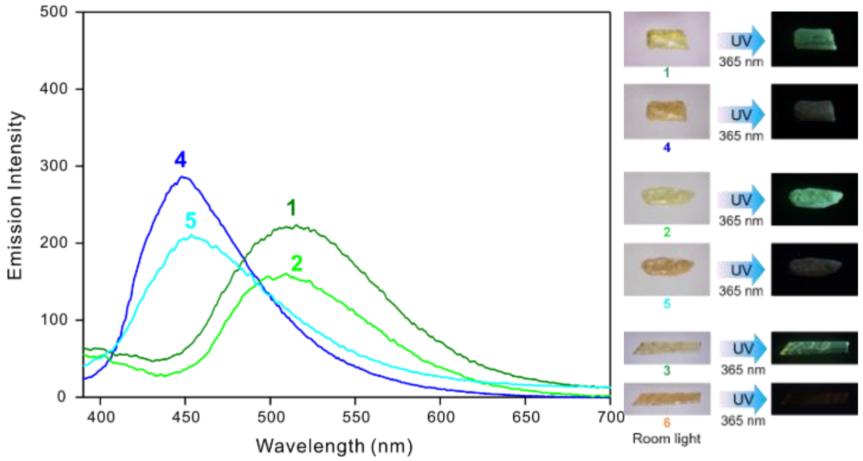

(a) (b)

Figure S17 Solid-state photoluminescence studies. (**a**) The PL spectra of **1**, **2**, **4**, and **5** at room temperature (excitation at 365 nm). The PL of **3** and **6** were not recorded due to lack of materials. (**b**) Images of single crystals under normal and UV lights. Crystal sizes for **1** and **4**: 0.30 mm × 0.58 mm × 0.24 mm; for **2** and **5**: 0.23 mm × 0.780 mm × 0.16 mm; for **3** and **6**: 0.19 mm × 1.03 mm × 0.35.

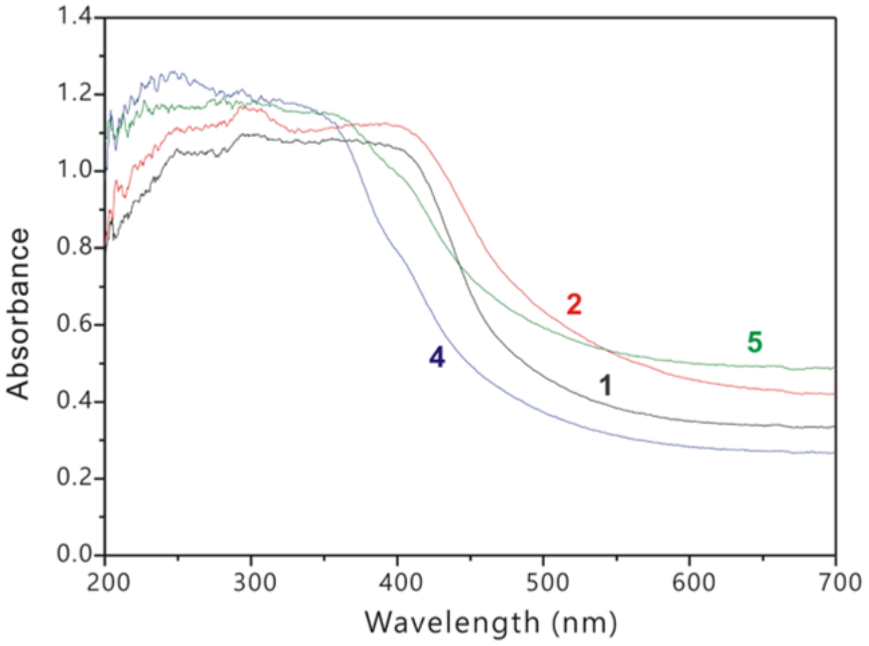

Figure S18 Solid-state UV-vis spectra of **1**, **2**, **4**, and **5**.

Isomerism in Coordination Polymers

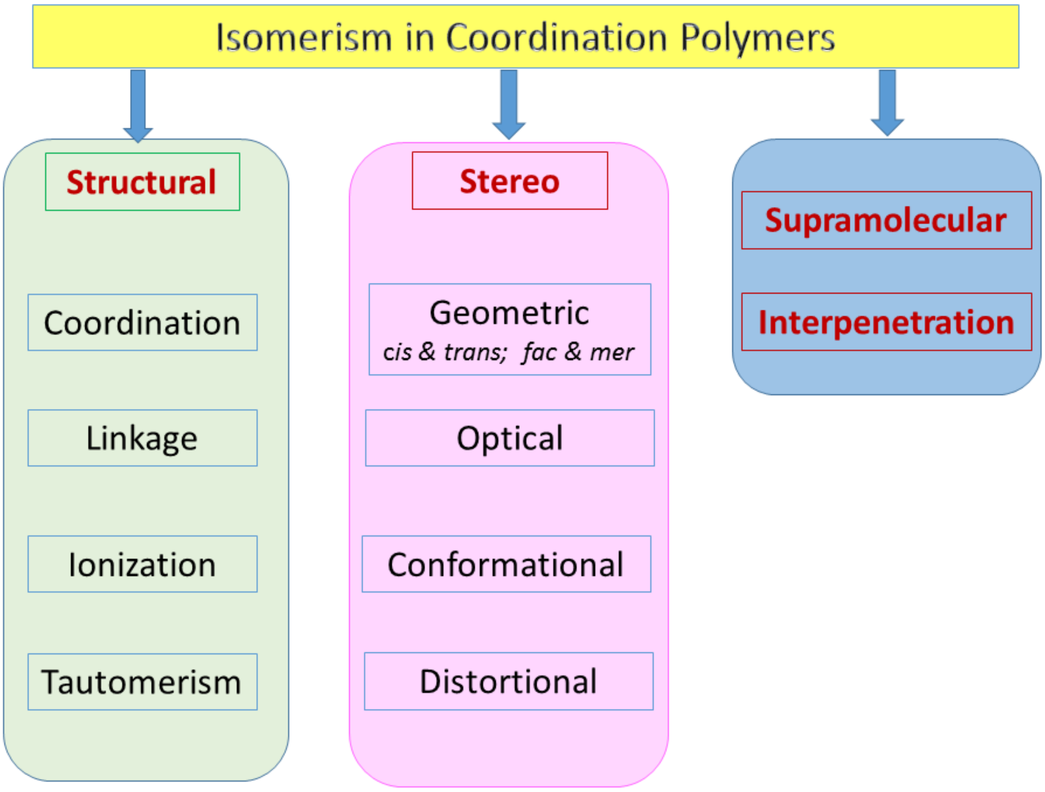

Depending upon the metal(II) dicarboxylate to bipyridyl spacer ligand ratio, two types of pillared-layer architectures are possible. For the metal(II) dicarboxylate to neutral dipyridyl spacer ligand ratio of 2:1 and 1:1, single pillared- and double pillared-layer structures can be generated (Fig. S19). Usually paddlewheel repeating units are found in single pillared-layer structure in which the metal(II) centre has square-pyramidal geometry, while dinuclear building blocks are observed for double pillared-layer structure with the metal(II) centres have octahedral geometries. The 1:1 ratio can also generate diamondoid topology.

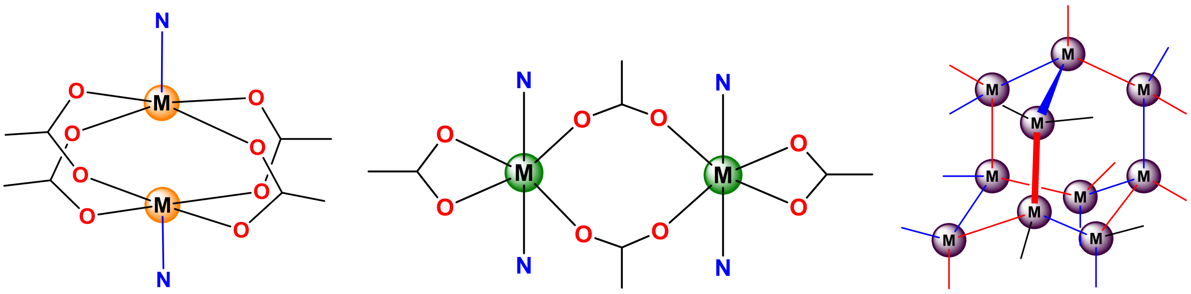

Figure S19 The basic building blocks for pillared-layer structures. (a) Paddlewheel repeating unit (Metal: dicarboxylate: dipyridyl spacer = 2:2:1 ratio) (b) Dinuclear repeating unit (Metal: dicarboxylate: dipyridyl spacer = 1:1:1 ratio) (c) Diamondoid connectivity.

In these pillared-layer 3D architectures, [M_2(dicarboxylate)$_2$] will form (4,4) grid layers and the nitrogen spacer ligands occupy the pillars to complete the **pcu** connectity in both the cases (Fig. S20).

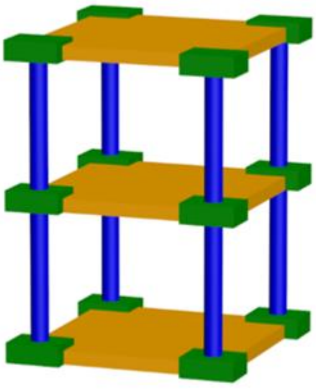

Figure S20 Single and double pillared-layer coordination polymers.

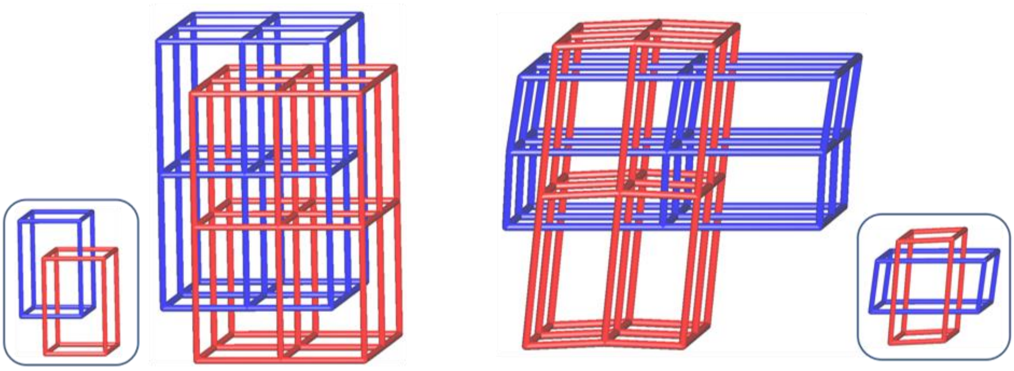

(a) (b)

Figure S21 **pcu** topology with (a) parallel interpenetration in **1** & **2** and (b) perpendicular interpenetration in **3**.

References

Balamurugan, S., Nithyanandan, S., Selvarasu, C., Yeap, G. Y. & Kannan, P. (2012). *Polymer*, **53**, 4104-4111

Elacqua, E., Bučar, D.-K., Skvortsova, Y., Baltrusaitis, J., Geng, M. L. & MacGillivray, L. R. (2009) *Org. Lett*. **11**, 5106-5109.

Spek, A.L. (2015) *Acta Cryst.* C71, 9-18

Horner, M., Hünig, S. (1982). *Liebigs Ann. Chem*., 1183-1210.

Peedikakkal, A. M. P., Vittal, J. J. (2008). *Chem. Commun*., 441-443.

Peedikakkal, A. M. P., Peh, C.S.Y., Koh, L.L., Vittal, J. J. (2010). *Inorg. Chem*., **49**, 6775-6777
